# **Extrapedicular approach for basivertebral nerve radiofrequency ablation: a conceptual review**

**DOI:** 10.1186/s13018-026-07074-x

**Published:** 2026-06-29

**Authors:** Jimmy Wen, Megan Kou, Nathaniel Duong, Adam Razick, Arsh Alam, Sugamjot Badhan, Jose Silva, Megan Hsu, Sam Warner, Rohin Thomas, Foad Elahi

**Affiliations:** 1 California Northstate University College of Medicine, 9700 W Taron Dr, Elk Grove, CA 95757 USA; 2 Department of Medicine, Highland Hospital, 1411 E 31st St, CA 94602 Oakland, USA; 3 University of California, Los Angeles, 405 Hilgard Avenue, Los Angeles, CA 90095 USA; 4 University of California, Davis, One Shields Avenue, Davis, CA 95616 USA; 5 Sutter Roseville, Emergency Medicine, 1 Medical Plaza Drive, Roseville, CA 95661 USA; 6 Touro University College of Osteopathic Medicine, 1310 Club Drive, Mare Island, Vallejo, CA 94592 USA; 7 Departments of Orthopaedic Surgery and Neurosurgery, Stanford University, Stanford, CA 94063 USA; 8 California Center of Pain Medicine & Rehabilitation, 4944 Sunrise Blvd, Fair Oaks, CA 95628 USA; 9 Davis, USA

**Keywords:** Basivertebral nerve, Radiofrequency ablation, Transpedicular, Extrapedicular, Low back pain

## Abstract

**Background:**

Chronic axial low back pain (LBP) of vertebrogenic origin is increasingly recognized as a distinct clinical entity responsive to basivertebral nerve (BVN) radiofrequency ablation (RFA). This analysis synthesizes the anatomical, biomechanical, and safety considerations of both access routes and evaluates their clinical implications.

**Methods:**

A literature review was conducted in PubMed for articles on BVNRFA anatomy, techniques, and access routes published through December 1, 2025. Given the limited literature on extrapedicular BVNRFA, studies from the vertebral augmentation literature were included if appropriate for access route considerations.

**Results:**

The standard technique for BVNRFA utilizes a transpedicular trajectory to access the vertebral body, which provides a consistent trajectory using the pedicle but places the introducer in proximity to the facet joints, dorsal root ganglion, exiting spinal nerve, and paraspinal muscles. Emerging evidence has described the extrapedicular approach, which follows a lateral pathway, may be considered for select patients with anatomical challenges, such as narrow pedicles or preexisting pedicle instrumentation.

**Conclusion:**

Currently, there is no clinical evidence to suggest the superiority of the extrapedicular approach over the traditional transpedicular approach. Each has its own anatomical considerations, technical challenges, and risks to consider, which require imaging guidance and careful adherence to anatomical landmarks. Further biomechanical, cadaveric, animal model, and clinical studies are warranted to assess the relative efficacy, safety, and long-term implications of each approach.

## Background

Vertebrogenic pain, characterized by nociceptive signaling from the vertebral endplates transmitted by the basivertebral nerve (BVN), has become more well-defined through clinical and radiographic correlation [1–3]. As a result, targeted intervention options now include basivertebral nerve radiofrequency ablation (BVNRFA) for patients with chronic low back pain (LBP) with Modic type 1 and 2 changes [4–6]. Anatomically, both vertebral endplates are innervated by the BVN through the center of the vertebral body. The pain transmitted from the vertebral endplate to the BVN is thus termed vertebrogenic pain, which is the basis of BVNRFA, where this pain transmission signal is interrupted via destruction of the BVN [7, 8]. The International Society for the Advancement of Spine Surgery (ISASS) policy statement summarized the existing evidence and recommended that chronic vertebrogenic pain (with Modic type 1 and 2 changes) is most successfully addressed by BVNRFA, with evidence showing a significant decrease in pain and improvement in function that is sustained > 5 years after treatment [9].

The traditional access to the vertebral body for BVNRFA involves traversing the pedicle, as the pedicle provides a radiographically visible and reproducible landmark. Additionally, traversing this osseous channel minimizes the risk of damaging the adjacent neurovascular and visceral structures. A perspective review reported that device or procedure-related complications ranged from 2.7% to 6.7%, with the most common being mild, transient leg pain or paresthesias that resolve within three months [10]. This transient leg pain is attributed to medial pedicle breach but is mild in nature and resolves on its own, requiring no interventions aside from a course of oral medications thus far [11–13]. Furthermore, out of 473 reported clinical procedures, there were two cases of vertebral compression fracture in a patient taking hormonal therapy, one case of retroperitoneal hemorrhage, and two device-procedure-related adverse events [1].

The pedicle is a major component of transmitting axial and mechanical load from the posterior wall of the vertebral body and posterior spinal elements such as the lamina and spinous process [14]. The biomechanical sequelae of violating the pedicle at this time are speculative. Therefore, pedicle integrity is a factor to consider, as traversing the pedicle does have a small risk of pedicle wall breach. Anatomical studies of the lumbar pedicle found that the height is greater than its width, and the upper and lower walls are composed of thicker cortical bone [15]. Thus, breach of the pedicle mainly occurs along the inner and outer walls rather than the upper and lower walls [16]. Screw penetration incidence is also closely related to the morphology of the pedicle, as the shape and pedicle axis angle differ by lumbar level [17, 18]. Another possible complication of pedicle violation as demonstrated by pedicle screw fixation is facet joint violation, which is associated with adjacent segment disease (ASD) due to altered spinal biomechanics, leading to increased load borne by the cranial facet joints [17–20]. However, these concerns with a 7-gauge trocar used for BVNRFA have not been substantiated.

**Fig. 1:**
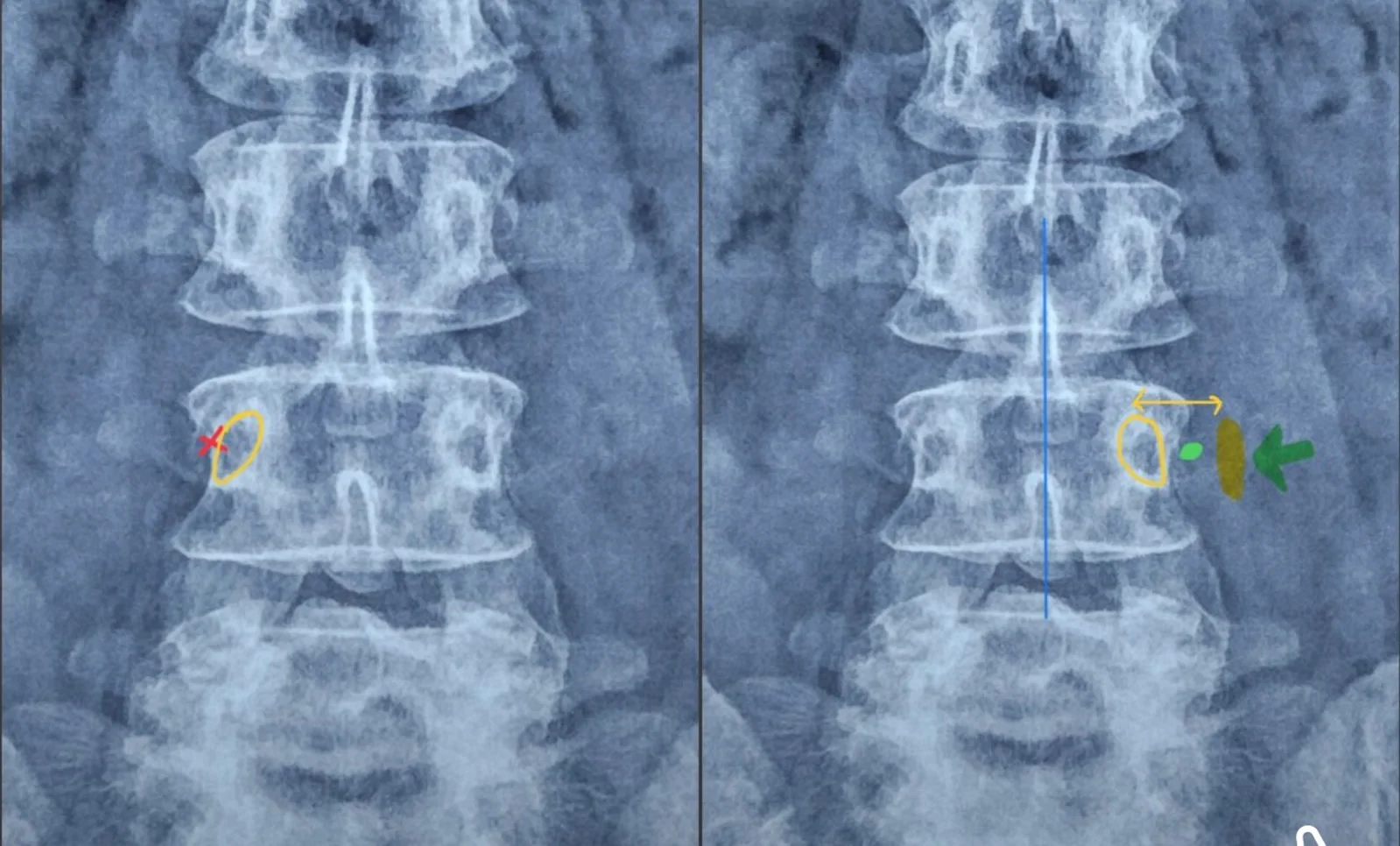
Fluoroscopic depiction of transpedicular versus extrapedicular approach

In contrast, the extrapedicular approach, where access to the posterolateral vertebral body is obtained lateral to the pedicle, preserves pedicle cortical integrity but increases the risk to surrounding structures (Fig. 1).

This conceptual review aims to explore the anatomy of both approaches, technical and safety considerations, clinical implications, and future directions for utilizing the extrapedicular approach as an alternative approach for BVNRFA. Given that no comparative trials currently exist comparing transpedicular versus extrapedicular approaches for BVNRFA, the available anatomical considerations are largely extrapolated from vertebral augmentation literature, which may not fully translate to BVNRFA.

### Literature search methods

A focused literature search was conducted in PubMed for articles on BVNRFA anatomy, techniques, and access routes published through December 1, 2025. The search strategy included the following keywords alone or in combination with MeSH terms (OR, AND): basivertebral nerve ablation, basivertebral nerve radiofrequency ablation, BVNRFA, BVNA, transpedicular, extrapedicular, pedicle, anatomy, vertebroplasty, kyphoplasty, vertebral augmentation. Given the limited literature specifically on BVNRFA, we included studies from the vertebral augmentation literature if appropriate for access route considerations.

### Transpedicular versus extrapedicular approach: anatomical considerations

Both the transpedicular and extrapedicular routes target a point 30 to 50% of the vertebral body from posterior to anterior, though their pathways differ in terms of anatomical navigation and safety risks [1, 20].

#### Facet joints

The facet joints are created by the articulation of the superior and inferior articular processes of adjacent vertebrae and are located posterior and slightly lateral to the pedicles. They work in conjunction with the intervertebral disc to provide structural stability and spinal mobility to the vertebral column [21, 22]. The close proximity between the pedicles and facet joints increases the risk that a missed trajectory can violate the facet capsule or superior articular process in the transpedicular approach. Injury to the facet joints can cause facet joint osteoarthritis, stiffness, and increased stress on adjacent segments [20]. Facet joint violation can range from mild symptoms such as localized tenderness and limited range of motion to more severe complications such as joint instability and accelerated degeneration [23]. Fluoroscopic or computed tomography (CT) image guidance is essential for accurate procedural trajectory and to avoid facet joint violation [22, 24]. The extrapedicular approach is further removed from the facet joint by using a trajectory lateral to the pedicle, theoretically decreasing the risk of facet joint violation [25].

#### Neurovascular structures

The neural foramina, which are bordered superiorly and inferiorly by adjacent pedicles, contain multiple neurovascular structures that are essential for neurologic function and can be placed at risk in the event of a pedicle breach during transpedicular BVNRFA [18]. Thus, understanding the relationship between these structures and the pedicle is paramount, as the risk and symptoms depend on which pedicle cortex is breached.

In the spinal canal, the dorsal and ventral nerve roots travel medial to the pedicle before joining to form the spinal nerve. A medial breach of the pedicle risks damage to the nerve roots and can result in radiculopathy and/or dysesthesias [1]. The dorsal roots also converge to form the dorsal root ganglion (DRG), which is located in the superior aspect of the neural foramina and inferior to the pedicles. This structure houses the primary sensory neuron cell bodies responsible for transmitting afferent information from peripheral tissues [26]. Thus, breaching the inferior border of the pedicle can cause direct trauma to the DRG, resulting in neuropathic pain and loss of sensory and autonomic function [27]. Although spinal cord injury is theoretically possible from BVNRFA, the conus medullaris terminates at L1-L2, and BVNRFA is Food and Drug Administration (FDA)-approved only at L3-S1; no spinal cord injuries have been reported in the literature [1]. Furthermore, there may be a risk of thermal injury and spread into the epidural space if the probe is placed near the medial pedicle wall or posterior vertebral cortex [1].

Additionally, each lumbar spinal nerve passes through the superior aspect of the neuroforamen and travels inferior and lateral to the pedicle at its corresponding level [18]. A cadaveric study found that the exiting spinal nerve lies 0.8–2.8 mm inferior to the pedicle, which leaves a narrow safety margin during transpedicular access techniques [28]. Therefore, inferior or medial breaches of the pedicle directly place the exiting spinal nerve at risk for damage, which may cause motor/sensory deficits [1]. With an extrapedicular approach, where a more lateral route is taken, the trajectory can be directed superior to the exiting spinal nerve at the targeted level, but careful navigation is required to avoid the descending spinal nerve from the level above, which courses along the anterolateral vertebral body. As the cannula is advanced with an extrapedicular approach towards the posterolateral vertebral body, careful navigation is required to avoid contacting the descending spinal nerve [25].

At each lumbar level, segmental lumbar arteries branch off the abdominal aorta and travel posterolaterally across the vertebral body and medial to the pedicle before giving off a dorsal branch. From the dorsal branch, a spinal/radicular branch enters the intervertebral foramen to supply the nerve root, dura, and DRG [29]. Lumbar veins accompany the arteries and include intervertebral veins that run along the margins of the pedicle [30]. These vessels may be affected by any lateral or inferior breaches of the pedicle, leading to bleeding or hematoma formation [26]. Thus far, the most severe vascular complication from BVNRFA reported in the literature has been retroperitoneal hemorrhage [8]. With an extrapedicular approach, there is a different vascular risk as the trajectory is more lateral, which brings the cannula closer to the dorsal branch and segmental arteries. Studies have demonstrated the feasibility of extrapedicular access at the L1 through L3 levels from the posterosuperior region of the vertebral body, as these regions are relatively avascular. Conversely, there is a greater variability of lumbar arteries and a relatively higher risk of lumbar artery injury with extrapedicular access at the L4 and L5 levels. This is clinically important as BVNRFA is most commonly performed at L5-S1, L4-L5, and L4-L5-S1, increasing the likelihood of encountering these variable arteries using the extrapedicular approach at these levels [7–8]. Thus, careful navigation is essential as these vessels course along the posterolateral vertebral body. These anatomical considerations highlight that both transpedicular and extrapedicular approaches have their own vascular risks.

#### Paraspinal muscles

The lumbar paraspinal muscles play a crucial role in moving and stabilizing the spine and include the iliocostalis, longissimus, and multifidus [31]. The multifidus is the deepest and most medial paraspinal muscle and originates from several structures, including the mamillary processes of the lumbar vertebrae, which are medial to the pedicles [31, 32]. Its positioning medial to the pedicle and directly posterior to the pars interarticularis and facet joint means that the thickest and most medial aspect of the multifidus is traversed by the needle and cannula during the transpedicular approach to BVNRFA, potentially leading to localized muscle inflammation and post-procedural discomfort [22]. The multifidus is segmentally innervated by the medial branch of the dorsal ramus of each corresponding spinal nerve, which allows it to provide control of small intervertebral motions at each segment [32–34]. Additionally, inadvertent contact with the medial branch may lead to secondary muscle atrophy or contribute to transient post-procedural localized pain or discomfort [35, 36]. However, an extrapedicular approach travels lateral to the facet and decreases the risk of violating the medial branch. These associations of multifidus atrophy and fibrosis, and worse postoperative pain and function outcomes have been demonstrated within studies of lumbar posterior surgery [37]. However, these findings are from significantly greater muscle dissection and tissue disruption than the 7-gauge trocar used during BVNRFA and should not be extrapolated as direct biomechanical evidence of similar effects following BVNRFA.

The erector spinae muscle group is more superficial to the multifidus and includes the iliocostalis and longissimus. The iliocostalis is the most lateral of the paraspinal three muscles, while the longissimus lies between the iliocostalis and the multifidus [38]. The erector spinae muscles are innervated by the lateral branches of the dorsal rami and play a key role in spinal extension, maintaining posture, and lateral flexion of the spine [39, 40]. The transpedicular approach primarily travels through the multifidus and longissimus, while the extrapedicular approach more commonly involves the erector spinae muscles [25, 38, 41]. The trajectory towards the BVN through the paraspinal muscles should be carefully studied in both transpedicular and extrapedicular BVNRFA.

#### Bone

Both approaches have their own theoretical considerations regarding bone integrity and risk. Pedicular breach is a potential risk in BVNRFA, and assessment of pedicle morphology preoperatively to minimize this risk. A commonly cited conservative threshold used to prevent pedicle breach is an 80% pedicle screw diameter-to-pedicle width ratio [42]. Additionally, maintaining a minimum cortical margin of 0.5 mm in the medial and lateral aspects of the pedicle can help prevent pedicle fracture [42]. Therefore, the selection of the side offering the greatest safety margin, based on width and cortical margin, is critical. If magnetic resonance imaging (MRI) shows an adequate pedicle diameter, no further imaging is necessary; however, if uncertainty exists, a CT scan or extrapedicular approach can be considered [42]. Therefore, careful evaluation of pedicular morphology is vital to minimize the risk of pedicular breach [42, 43]. Transpedicular access requires transgressing the pedicle, raising theoretical concerns about decreased pedicle integrity, but the long-term biomechanical data for a single-cannula defect with BVNRFA are currently lacking [14]. In contrast, the extrapedicular approach creates a posterolateral vertebral tract, whose biomechanical implications remain unknown. Notably, the vertebral body is vulnerable to compression fractures as bone mineral density decreases with age. Bone mineral density is also positively correlated with the biomechanical properties of lumbar vertebrae [44]. Theoretically, if cortical integrity is affected with a posterolateral tract, changes in vertebral biomechanics may occur. However, the risk of vertebral compression fractures and/or altered load transfer to other spinal elements has not been demonstrated in BVNRFA and remains unknown. Another consideration for the extrapedicular approach is that a lateral trajectory increases the risk of transgressing the transverse process. The lumbar spine also varies between individuals, with these congenital variations being known as lumbosacral transitional vertebrae [45, 46]. Notably, in patients without these variations, the longest transverse process is located at L3 [45, 46]. The 7-gauge trocar used in BVNRFA is larger than the one used for augmentation, further increasing the risk of contacting cortical bone [47]. Although this hasn’t been reported in the literature, the anatomical basis warrants caution when planning the trajectory. However, this is an anatomically possible concern, especially as some of these variations require contacting or traversing against the transverse process, as we discuss their trajectories later.

**Fig. 2:**
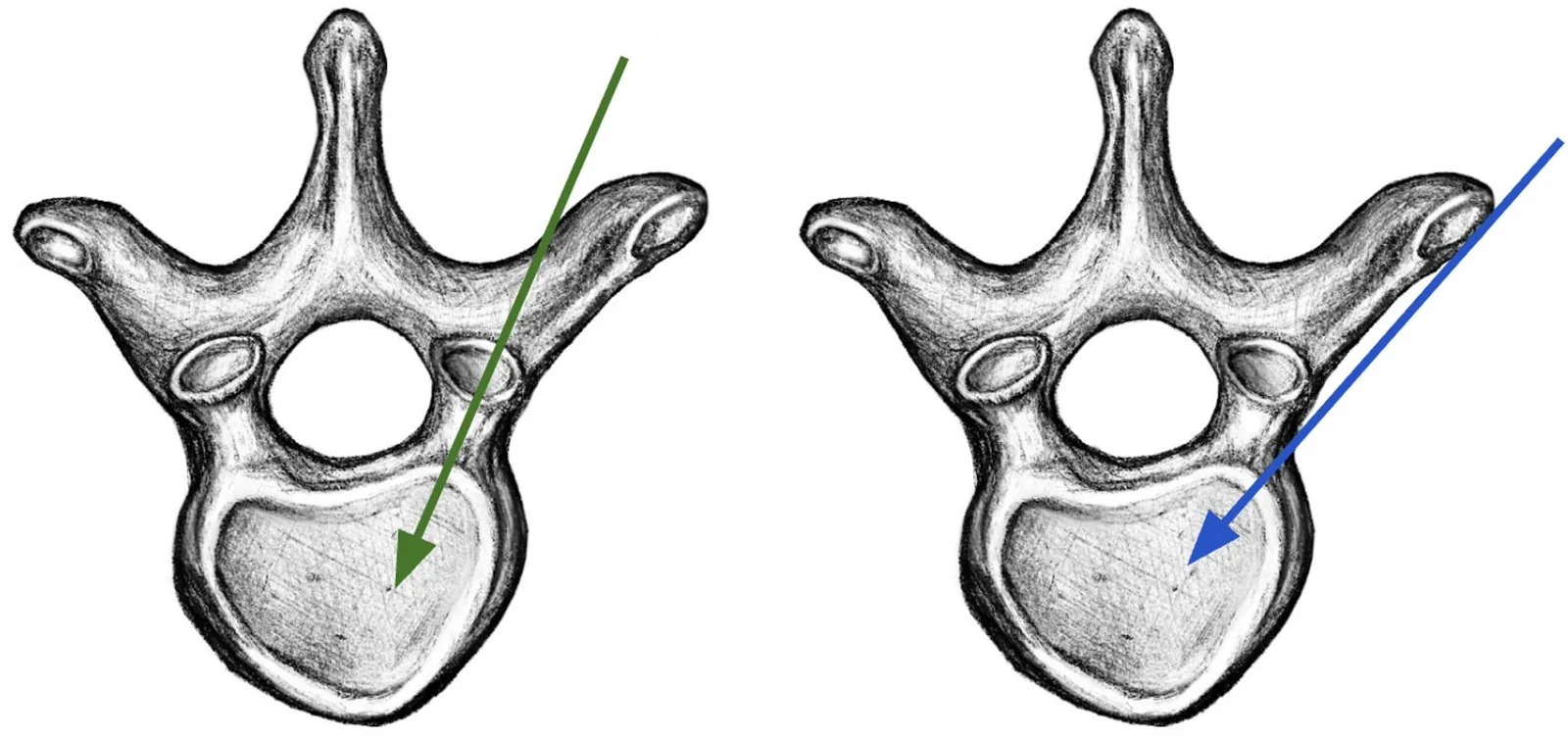
Axial cross-section comparing transpedicular versus extrapedicular approach. Transpedicular (left); Extrapedicular (right)

A comparison between the transpedicular and extrapedicular approaches can be found in Table 1, and an axial depiction in Fig. 2.

**Table 1:** Transpedicular versus Extrapedicular relationship with adjacent structures

Variable	Transpedicular	Extrapedicular
Vascular injury	Low risk overall, as the segmental arteries are more lateral to the pedicle, though retroperitoneal hemorrhage has been reported	Increased risk at L4-L5 due to the proximity of the segmental arteries
Pedicle integrity	Transgressed	Preserved
Facet joint	Possible if a too medial approach	Typically lateral to the facet joint
Muscle	Medial entry through multifidus	Lateral entry through the erector spinae
Spinal nerve	Usually safe if within pedicle walls	Requires careful angulation to stay superior to the spinal nerve
Vertebral body access	Central	Oblique towards the posterolateral vertebral body
Long-term implications	Single-tract pedicle transgression does not have long-term biomechanical data to confirm its influence on future instrumentation	Preserves the pedicle, but no evidence that this approach has a positive effect if future fixation is required. Long-term biomechanical data on creating a posterolateral tract are unavailable.

### Overview of transpedicular and extrapedicular approaches

#### Transpedicular approach 

In the traditional transpedicular technique of BVNRFA, the pedicles serve as critical anatomical landmarks and as access routes to the vertebral body [22]. Beginning at the pedicle’s superior lateral aspect, the trocar is passed carefully along the pedicle with the use of a mallet until it reaches the posterior vertebral body. As the trocar is advanced, it is crucial to maintain a trajectory superior to the inferior cortex and lateral to the medial cortex to prevent entry into the spinal canal or adjacent neural elements [1]. Once vertebral body access is obtained, the trocar is removed from the introducer cannula and replaced with a curved cannula (with a curved stylet). This allows a shaping of a curved channel towards the target site at the BVN terminus at the midline [1].

#### Extrapedicular approach

More recently, vertebral body access via an extrapedicular approach has been described, but mainly for vertebral augmentation, rather than BVNRFA. An extrapedicular approach may be considered if pedicle width is too narrow for the trocar on either side, in the instance of preexisting pedicle screws, if there is vasculature in the trajectory of pedicular access, and in patients with very narrow pedicles (pedicle angulation significantly less than 30 degrees), as this can increase the risk of pedicular violation [42]. In cases of ASD with prior pedicle screw instrumentation, BVNRFA can be accomplished by screw removal followed by a transpedicular approach. Avoiding screw removal is often preferred to avoid surgical risks; therefore, an extrapedicular approach would be considered a reasonable option in such cases [25].

Several variations of this technique may be selected based on the physician’s preference and patient-specific anatomy.

**Fig. 3:**
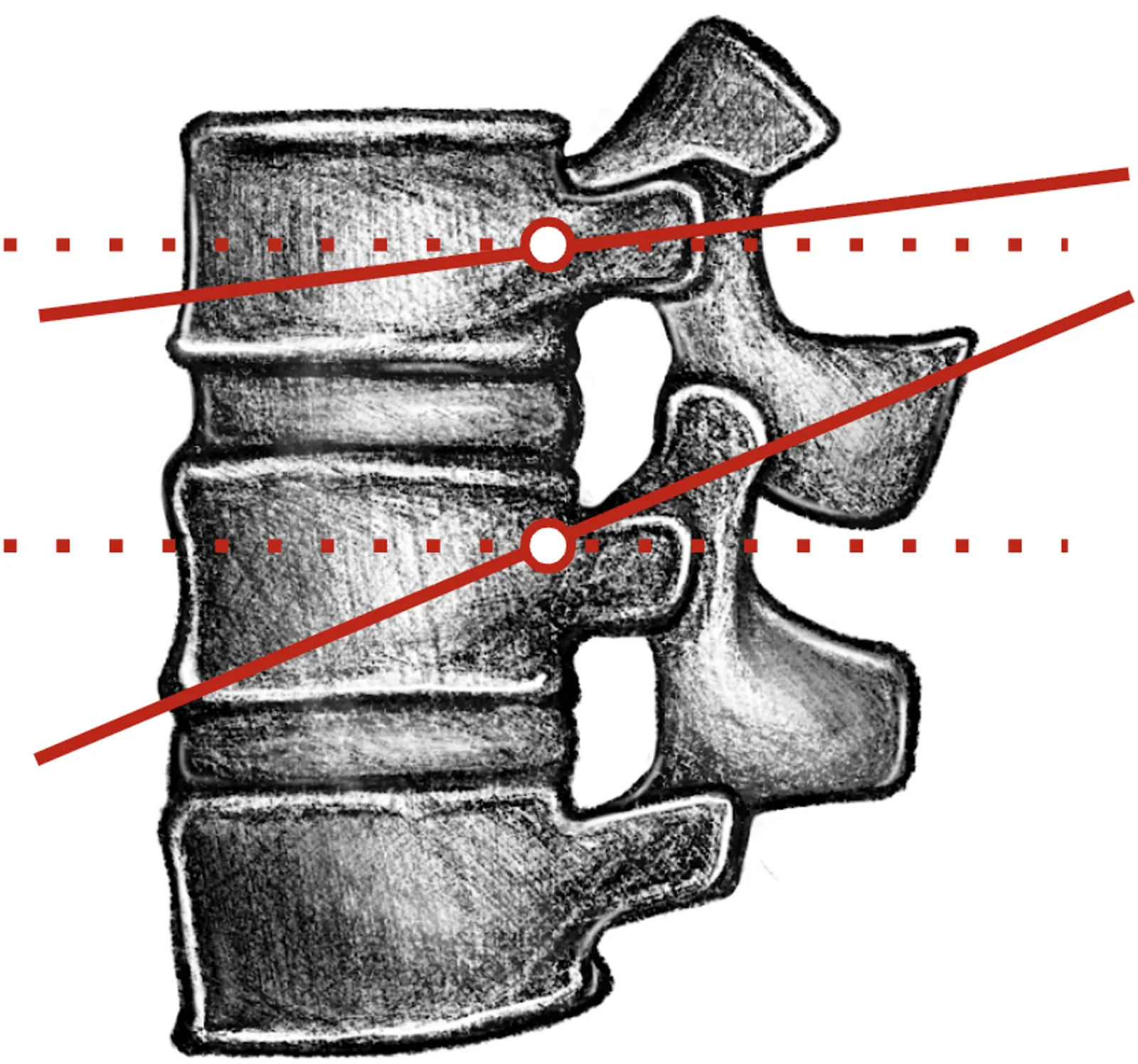
Sagittal schematic of entry points and target zones for transpedicular and extrapedicular. Above: transpedicular, Below: extrapedicular

The introducer needle is positioned 0.5–1 cm lateral to the pedicle and the middle/upper aspect of the transverse process [48]. Some physicians prefer starting at the superior lateral edge of the pedicle with varying angulation depending on the pedicle level. Before the procedure, the inferior and medial walls of the pedicle must be visualized to minimize the risk of breaching into the neuroforamina or spinal canal [48–50]. The introducer cannula is advanced through the soft tissue lateral to the pedicle and directed medially toward the posterolateral wall of the vertebral body (Fig. 3).

In the following subsections, variations of the extrapedicular approach are described using vertebral augmentation (Table 2), as there is no direct literature on these approaches in BVNRFA.

**Table 2:** Definitions of the various extrapedicular approaches

Approach	Definition
Extrapedicular	Broad term encompassing approaches that access the vertebral body without traversing the pedicle
Parapedicular	Directed adjacent to the pedicle and typically overlying the transverse process
Far-lateral extrapedicular	More lateral entry point than conventional extrapedicular approaches, directed beyond the transverse process and advanced along the pedicle
Unilateral extrapedicular	Extrapedicular approach directed lateral to the transverse process, avoiding bilateral instrumentation
Extrapedicular modified inferior endplate	Modified extrapedicular approach directed above the inferior endplate and anterior to the pedicle
Kambin triangle	Extrapedicular approach using Kambin’s triangle as a safe corridor and directed towards the superolateral area of the target endplate
Superior pedicle	Extrapedicular approach directed superior to the pedicle rather than lateral to it
Modified extrapedicular with pedicle screws and adjacent segment disease	Modified extrapedicular approach to accommodate and navigate around preexisting pedicle screw instrumentation

#### Parapedicular approach

**Fig. 4:**
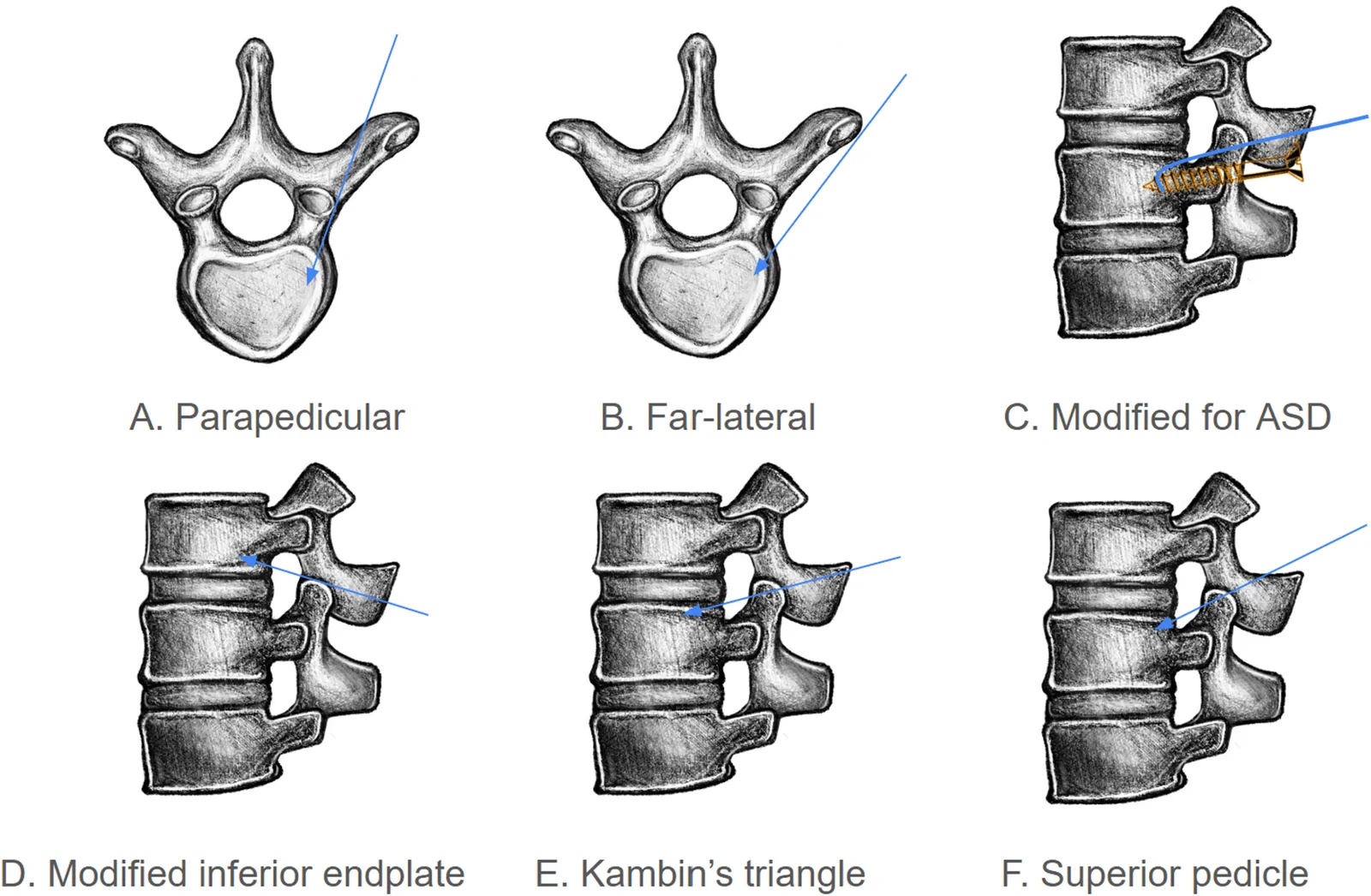
Variations of the extrapedicular approach.** A**: parapedicular approach;** B**: far lateral approach;** C**: modified extrapedicular with pedicle screws and adjacent segment disease with J stylet curving inferiorly over the pedicle screw;** D**: modified inferior endplate approach;** E**: Kambin’s triangle approach;** F**: superior pedicle approach

A cadaveric study consisting of 102 vertebral compression fractures from T4 to L5 was treated using a “parapedicular” approach [51]. The authors found, during the dissection, a relatively avascular and aneural area along the superior margin of the vertebral body-pedicle junction. The procedures were all completed without recognized clinical complications from the needle access or instrumentation [51]. Zhuo et al. utilized the extrapedicular approach on 101 fractured vertebrae without any clinical complications [52]. Their route involved locating and overstriding the transverse process until the needle reached the lateral cortex of the pedicle (Fig. 4). The craniocaudal and extraversion angle of the needle is adjusted as needed until it sticks into the depressed bone groove at the superolateral junction of the pedicle/vertebral transitional location. At this penetration point, the bone drill was inserted and advanced until the anterior cortex of the vertebral body was reached, before a working cannula was inserted to inject bone cement [52].

These techniques are shown as a conceptual spectrum of extrapedicular access routes,* with illustrations of the variations in entry points and trajectories.*

#### Far-lateral extrapedicular approach

Ryu et al. utilized a far-lateral extrapedicular approach among 31 patients [53] and 27 patients [54] with thoracic and lumbar vertebral compression fractures. This approach involves a skin insertion at 1 cm outside the costotransverse joint (thoracic vertebrae) or 1 cm lateral to the lateral one-third of the transverse process (lumbar vertebrae). The puncture needle’s transverse section angle is maintained at 45–50 degrees, advancing along the direction of the pedicle (Fig. 4). When the needle reaches the costotransverse joint (thoracic vertebrae) or the transverse process (lumbar vertebrae), the needle is hammered into the posterolateral vertebral body cortex with a target location at the junction of the anterior vertebral body cortex and lower endplate of the target vertebrae [53, 54].

#### Unilateral extrapedicular approach

With this approach, the puncture incision is located 1–3 cm lateral from the tip of the transverse process, and the puncture needle is passed through the superior margin of the medial one-third of the transverse process. The needle is then advanced until the needle tip is placed at the junction between the superolateral part of the pedicle and the vertebral body. Once the cortex is penetrated, the needle may require slight adjustment to direct the needle into the target midline of the vertebral body [55].

For the lumbar extrapedicular approach, the skin incision point varied depending on the level. The insertion point for L1, L2, L3, and L4 is 8 cm from the midline, 9 cm, 10 cm, and 11 cm, respectively. However, the iliac crest prevents this approach at the L5 level. Thus, the needle is navigated at a transverse section angle of 60 degrees to pass in front of the transverse process to reach the junction of the pedicle and the posterior wall of the vertebral body [56].

#### Extrapedicular modified inferior endplate approach

Beall et al. utilized this approach for thoracolumbar access for 96 vertebral compression fractures from T5 to L5. The skin puncture point and entry point into the vertebral body are approximately 0.5–1.0 cm above the lower endplate and in front of the pedicle (Fig. 4). The puncture needle is maintained parallel to the horizontal plane and guided towards the middle of the front one-third of the vertebral body [57].

#### Kambin triangle approach

Wang et al. evaluated this approach for 144 procedures from T6 to L5. The skin puncture point is 5 to 7 cm from the midline at the inferior endplate of the superior normal vertebral body. The target is the superolateral area of the responsible endplate (Fig. 4), not beyond the medial border of the pedicle, with the final needle destination at the anterior one-third of the vertebral body [58].

#### Superior pedicle approach

Jiang et al. compared a superior pedicle approach versus the transpedicular control group and reported significantly lower rates of paravertebral leakage, shorter operative time, fewer fluoroscopies, and no lumbar artery injury in the superior pedicle group [59]. The rationale for this approach compared to the traditional extrapedicular route via the lateral border of the transverse process was its anatomical relationship to segmental arteries [60]. More recent studies have found that the border between the lateral border of the posterior superior margin of the vertebral body and the superior lateral pedicle is a relatively safe bone entry point (Fig. 4) [55, 61]. The advantage of this approach for vertebral augmentation is that a unilateral extrapedicular approach can provide bilateral symmetric cement dispersion. A unilateral transpedicular approach would not be able to do this due to the anatomic characteristics of the pedicle; thus, typically, a bilateral transpedicular approach is done to prevent asymmetric distribution of bone cement.

#### Modified extrapedicular approach with pedicle screws and ASD

Stolzenberg et al. describe a modified extrapedicular approach for patients in the setting of ASD, but several unique challenges must be navigated with this modification [25]. Firstly, preoperative sagittal MRI/CT imaging should demonstrate a spacious enough foramen directly above the targeted vertebral body to allow for docking below the spinal nerve (Fig. 4). Thus, foraminal stenosis that is severe enough to prevent a 4 mm introducer cannula from entering below the spinal nerve is a contraindication. The final probe location should be at least 1 cm from any screw to reduce the risk of thermal energy transfer to adjacent hardware. The introducer cannula assembly is first docked at the pedicle-vertebral body junction between the pedicle screw and the superior endplate. It is then advanced medially over the top of the screw and then caudally to reach the vertebral body. The J-stylet must be navigated through this trajectory before the J-stylet starts to turn inferiorly, while still aiming to remain in the posterior half of the vertebral body and ultimately reaching a midline target [25]. However, this technique has been described in only one published study by Stolzenberg et al. and has not been independently replicated or evaluated in larger cohort studies.

## Discussion

### Clinical implications

Currently, there are no comparative studies for the transpedicular versus extrapedicular approach for BVNRFA, and thus, vertebral augmentation literature was discussed for access route considerations. Although vertebral augmentation and BVNFA share a similar access route into the vertebral body, these two procedures differ substantially in instrumentation (delivery of cement versus creating access for ablation), trajectory goals, working cannula characteristics (cement injection versus lesioning), and procedural biomechanics (stabilization and load redistribution versus ablation without volumetric augmentation). A key limitation of this current review is that the approaches presented from vertebral augmentation literature may not fully translate to BVNRFA and should be interpreted as a conceptual framework rather than extrapolated as an evidence-based comparison.

Choosing between transpedicular and extrapedicular approaches requires considerations of anatomical feasibility, clinician experience, and individualized comorbidities rather than theoretical structural sequelae. Although the transpedicular route transgresses the pedicle, no current evidence in the literature demonstrates a long-term effect on pedicle integrity or future ability to sustain instrumentation. Additionally, based on principles of fracture healing and Wolff’s law, bone undergoes continuous remodeling in response to mechanical loading, and localized cortical defects may heal through these physiological reparative processes [62, 63]. However, this has not been evaluated or demonstrated in clinical, cadaveric, or biomechanical studies, and extrapolation to BVNRFA is a plausible but unverified hypothesis. Regarding future surgical options, a pooled analysis of three trials found at five-year follow-up, a substantial reduction in healthcare utilization rates such as conservative care, opioids, lumbosacral spinal injection, lumbosacral radiofrequency ablation, and lumbosacral fusions [64]. This suggests that theoretical concerns about compromising future surgical options are currently minimal. While pedicle screw studies with spinal fixation have demonstrated that cortical breaches result in reduced insertional torque and pullout strength and increased risk of fracture, this extrapolation towards BVNRFA is theoretical, as a BVNRFA cannula disrupts the pedicle to a lesser extent [65–67].

In contrast, the extrapedicular approach allows for preservation of the pedicle but also comes with its own theoretical considerations. Although current evidence is limited, an extrapedicular approach may be considered in certain anatomical considerations such as narrow pedicle width, preexisting pedicle screw instrumentation, unfavorable pedicle angulation, difficult sacral access, concern for medial pedicle breach, or other clinical situations where a transpedicular trajectory can be technically challenging (Table 3). Given the FDA-approved BVNRFA levels of L3-S1, the extrapedicular approach is likely the most feasible at L3 and L4, where the pedicles are wider and provide the most favorable anatomical corridor. L5 is not as anatomically feasible given the iliac crests obstructing the lateral trajectory, and S1 does not currently have a well-defined extrapedicular access point.

**Table 3:** Clinical scenarios where an extrapedicular approach may be considered

Clinical scenario	Rationale for considering an extrapedicular approach
Narrow pedicle morphology	May limit safe transpedicular access
Prior pedicle instrumentation	Preexisting hardware may prevent transpedicular access
Unfavorable pedicle angulation	Alternative trajectories may facilitate access to the vertebral body
Difficult sacral access	Sacral anatomic constraints may be technically challenging
Concern for medial pedicle breach	May reduce the risk of medial pedicle wall violation

The posterolateral access point of the vertebral body may theoretically compromise cortical integrity, but whether this increases the risk of vertebral compression fractures and/or alters load transfer to other elements of the spine remains unknown. Additionally, the larger introducer used in BVNRFA compared to vertebral augmentation may theoretically increase the risk of transgressing the transverse process. Similar to the theoretical concerns for the transpedicular approach, these remain unproven. Both approaches have their own benefits and actual/theoretical risks, and a balanced discussion is essential until long-term data is available. Overall selection of the approach requires careful imaging guidance, trajectory planning, and consideration of patient anatomy, pedicle morphology, and balancing of approach-specific risks.

### Future directions

Further biomechanical studies are necessary to determine if the transpedicular approach to BVNRFA carries the same risks as related pedicular surgeries. While current insights into the consequences of cortical disruption are based on pedicular screw studies, more research is needed to evaluate single-cannula defects from transpedicular BVNRFA. Additionally, current studies have not evaluated whether transgression of a pedicle will lead to a weaker, unchanged, or stronger bone over time. We recommend using animal models, as this can help assess healing and remodeling over specific time frames. Furthermore, mechanical testing, such as cadaveric loading models and finite-element simulations, can quantify how single-needle cortical defects affect vertebral biomechanics and future instrumentation. Without this data, the long-term structural impact of transpedicular access is unknown.

Moreover, further prospective clinical studies are essential to determine if the theoretical benefits of the extrapedicular over the transpedicular approach translate into practice. Primary outcomes such as pain reduction, opioid utilization, escalation of care, and duration of symptom relief should be measured. Additionally, secondary endpoints such as evidence of paraspinal, facet joint, and neural/vascular damage should be compared between the approaches.

Finally, the practical application of the extrapedicular route requires standardized procedures and protocols, such as imaging landmarks and safety corridors, for clinical adoption. Several extrapedicular variations discussed in this review, especially the modified extrapedicular approach with pedicle screws and ASD, have limited published studies. Further multicenter clinical studies are needed to determine which techniques are reproducible and suitable for specific situations/settings. Overall, standardized protocols would allow for consistent outcomes across a variety of practice settings. Future biomechanical and clinical studies of the extrapedicular approach, with the development of standardized protocols, are crucial to determine its practical benefits and ensure reproducible results.

## Conclusion

Currently, no clinical evidence has demonstrated the superiority of the extrapedicular approach over the traditional transpedicular approach for BVNRFA. Each approach has its own anatomical considerations, technical challenges, and risks to consider, which require imaging guidance and careful adherence to anatomical landmarks. The transpedicular approach offers a consistent trajectory and access point using the pedicle. Anatomical considerations suggest the extrapedicular approach may be considered in select clinical situations, such as patients with narrow pedicles or preexisting pedicle instrumentation. Currently, no long-term biomechanical data exist to determine if either approach results in meaningful alterations in structural or cortical integrity of the pedicle or vertebral body. The determination of approach should ultimately be individualized based on patient anatomy, clinician experience, and individualized comorbidities. Further studies, including biomechanical, cadaveric, animal models, and clinical research, are needed to assess relative efficacy, safety, and long-term implications.

## Data Availability

No datasets were generated or analysed during the current study.

## Abbreviations

BVN: Basivertebral nerve
BVNRFA: Basivertebral nerve radiofrequency ablation
LBP: Low back pain
ISASS: International Society for the Advancement of Spine Surgery
ASD: Adjacent segment disease
CT: Computed tomography
DRG: Dorsal root ganglion
MRI: Magnetic resonance imaging
FDA: Food and Drug Administration
